# Renal dioctophymosis in a free-ranging lesser grison (*Galictis cuja*): antemortem diagnosis and nephrectomy

**DOI:** 10.1007/s11259-026-11517-5

**Published:** 2026-09-16

**Authors:** Daniele Gehres, Bianca Cherem Corni, Pedro Almeida Rezende, Crisiele Junges Ramgrab, Julia Somavilla Lignon, Fabiane de Holleben Camozzato Fadrique, Laís Formiga Silva, Claudia Giordani, Fabiane Borelli Grecco, Josaine Cristina da Silva Rappeti, Raqueli Teresinha França

**Affiliations:** 1 Residency Program in Wildlife Medicine, Federal University of Pelotas, Pelotas, Rio Grande do Sul Brazil; 2 Residency Program in Preventive Veterinary Medicine, Federal University of Pelotas, Pelotas, Rio Grande do Sul Brazil; 3 Department of Veterinary Clinics, Faculty of Veterinary Medicine, Federal University of Pelotas, Pelotas, Rio Grande do Sul Brazil; 4 Veterinary Hospital, Faculty of Veterinary Medicine, Federal University of Pelotas, Pelotas, Rio Grande do Sul Brazil; 5 Department of Animal Pathology, Faculty of Veterinary Medicine, Federal University of Pelotas, Pelotas, Rio Grande do Sul Brazil

**Keywords:** Mustelidae, Wildlife medicine, Renal helminthiasis, Urinalysis, Abdominal ultrasonography, Wildlife release

## Abstract

Dioctophymosis is caused by *Dioctophyme renale*, a nematode that primarily affects the right kidney of domestic and wild mammals. This report describes the antemortem diagnosis, surgical treatment, and rehabilitation of a free-ranging lesser grison (*Galictis cuja*) infected with the parasite. An adult male weighing 1.85 kg was admitted after being struck by a vehicle and showed signs consistent with traumatic brain injury. During hospitalization, fecal parasitological examination revealed eggs consistent with *D. renale*, along with other parasitic structures. Urinalysis revealed mild proteinuria, hematuria, leukocyturia, marked bacteriuria, and numerous *D. renale* eggs. Abdominal ultrasonography showed multiple tubular and circular structures in the right kidney region, consistent with parasites, with no residual renal parenchyma identified. The left kidney was enlarged and showed mild changes in echogenicity. Given the extent of renal involvement, a right nephrectomy was performed, resulting in the removal of eight adult *D. renale* specimens, comprising seven females and one male. Histopathological examination confirmed complete destruction of the renal parenchyma, absence of viable glomeruli and tubules, and osseous and squamous metaplasia of the renal capsule. Serial hematological and biochemical evaluations showed no progressive increase in urea or creatinine concentrations. Postoperative recovery was uneventful, allowing the animal to recover and return to the wild. This case highlights the importance of combining urinalysis, ultrasonography, and clinical evaluation for the diagnosis and therapeutic planning of dioctophymosis in wild mustelids.

## Background

Dioctophymosis is caused by *Dioctophyme renale*, commonly known as the “giant kidney worm,” a parasite that predominantly infects the right kidney, but it may also occur in the abdominal cavity and other ectopic sites in domestic and wild mammals (Trindade et al. 2018; Eslahi et al. 2021; Assis-Silva et al. 2025). The generalist and opportunistic diet of wild carnivores may increase exposure to trophically transmitted parasites, particularly through the ingestion of prey that serve as intermediate or paratenic hosts (Pedrassani et al. 2009; Mascarenhas and Müller 2015; Mascarenhas et al. 2018, 2019). In wild hosts, *D. renale* has been identified during surgical procedures, as reported in South American coatis (*Nasua nasua*) (Milanelo et al. 2009), and during postmortem examinations, including in Geoffroy’s cats (*Leopardus geoffroyi*) and Pampas foxes (*Lycalopex gymnocercus*) (Trindade et al. 2018; Facelli Fernández et al. 2024), as well as in parasitological surveys of road-killed wild canid carcasses (Eslahi et al. 2021).

In North America, the American mink (*Neogale vison*) is recognized as an important natural definitive host of *D. renale* and contributes to the persistence of the parasite in wild environments (Mech and Tracy 2001; Mech 2008). In Brazil, infection has been reported in a Neotropical otter (*Lontra longicaudis*) in Rio Grande do Sul (Echenique et al. 2018) and in lesser grisons (*Galictis cuja*) in the states of Minas Gerais (Costa Silva et al. 2025), Paraná (Zabott et al. 2012), Santa Catarina (Pedrassani et al. 2017), and Rio Grande do Sul (Pesenti et al. 2012; Schwantes et al. 2020).

Although *G. cuja* is widely distributed throughout South America (Poo-Muñoz et al. 2014), published records of dioctophymosis in this species are based predominantly on postmortem findings from animals found dead, often after vehicle collisions (Barros et al. 1990; Pesenti et al. 2012; Zabott et al. 2012; Pedrassani et al. 2017; Schwantes et al. 2020). Information on antemortem diagnosis, treatment, and rehabilitation of affected individuals therefore remains limited. In this context, the present report aims to describe the diagnostic approach, surgical treatment, clinical course, and recovery of a lesser grison infected with *D. renale* in the right kidney.

## Case presentation

An adult male *G. cuja*, weighing 1.85 kg, was admitted to the Wildlife Rehabilitation Center and Wildlife Screening Center of the Federal University of Pelotas after being struck by a vehicle, in an urban area of Capão do Leão, Rio Grande do Sul, Brazil. On initial clinical examination, the animal showed signs consistent with traumatic brain injury (TBI), and treatment for this condition was initiated.

**Fig. 1:**
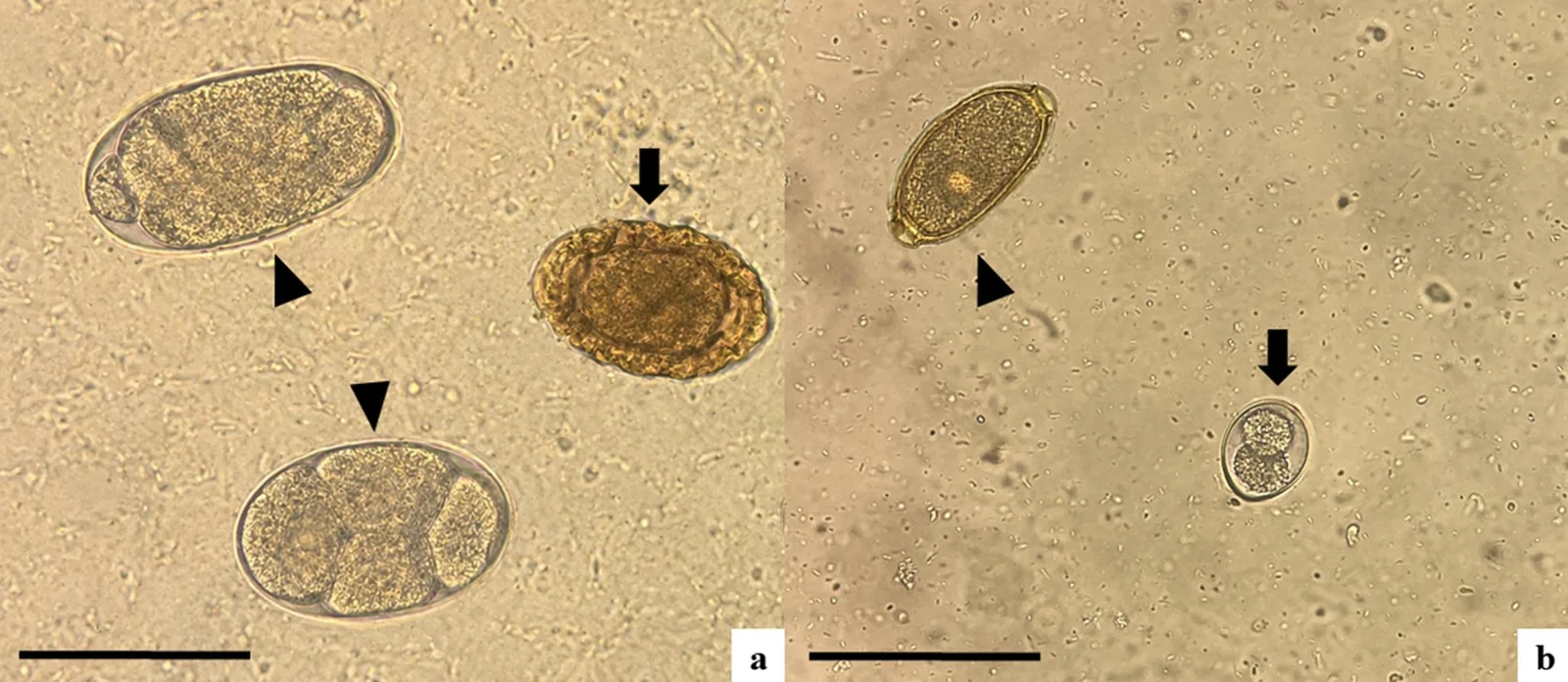
Parasitic structures observed during fecal parasitological examination of *Galictis cuja*. **a** Strongylida-type eggs (arrowhead) and a *Dioctophyme renale* egg (arrow). **b** Capillariid-type egg (arrowhead) and an unsporulated coccidian oocyst morphologically compatible with *Cystoisospora* spp. (arrow). Scale bar = 60 μm

During hospitalization, a fecal parasitological examination was performed on a sample collected from the enclosure (Fig. 1). The zinc sulfate centrifugal flotation technique, as modified by Monteiro (2017), revealed capillariid-type eggs, eggs morphologically consistent with *D. renale*, and Strongylida-type eggs, as well as unsporulated coccidian oocysts morphologically compatible with *Cystoisospora* spp. Eggs and oocysts were identified based on their morphological and morphometric characteristics according to published parasitological descriptions (Monteiro 2017; Pedrassani and Nascimento 2015; Lignon et al. 2026), using an Olympus optical microscope (CX22 series; Olympus Corporation, Tokyo, Japan).

Based on the coproparasitological findings, urinalysis was performed as described by Chew and Schenck (2023). The sample was collected by spontaneous urination during the physical examination. On gross examination, the urine was dark yellow and turbid, with a specific gravity of 1.042. Chemical analysis was performed using commercial reagent strips (Urina Sensi 10^®^, Sensitive^®^/CRAL, registration no. 10310030175) per the manufacturer’s instructions. Urinary pH was 6.5, urobilinogen was within the normal range, and bilirubin, occult blood, glucose, and ketone bodies were not detected. Mild proteinuria (+; 30 mg/dL) was observed. Urine sediment examination revealed 50–100 erythrocytes per high-power field (HPF), 50–100 leukocytes/HPF, 2–5 renal epithelial cells/HPF, marked bacteriuria (+++), spermatozoa (+++), and *D. renale* eggs (+++).

**Fig. 2:**
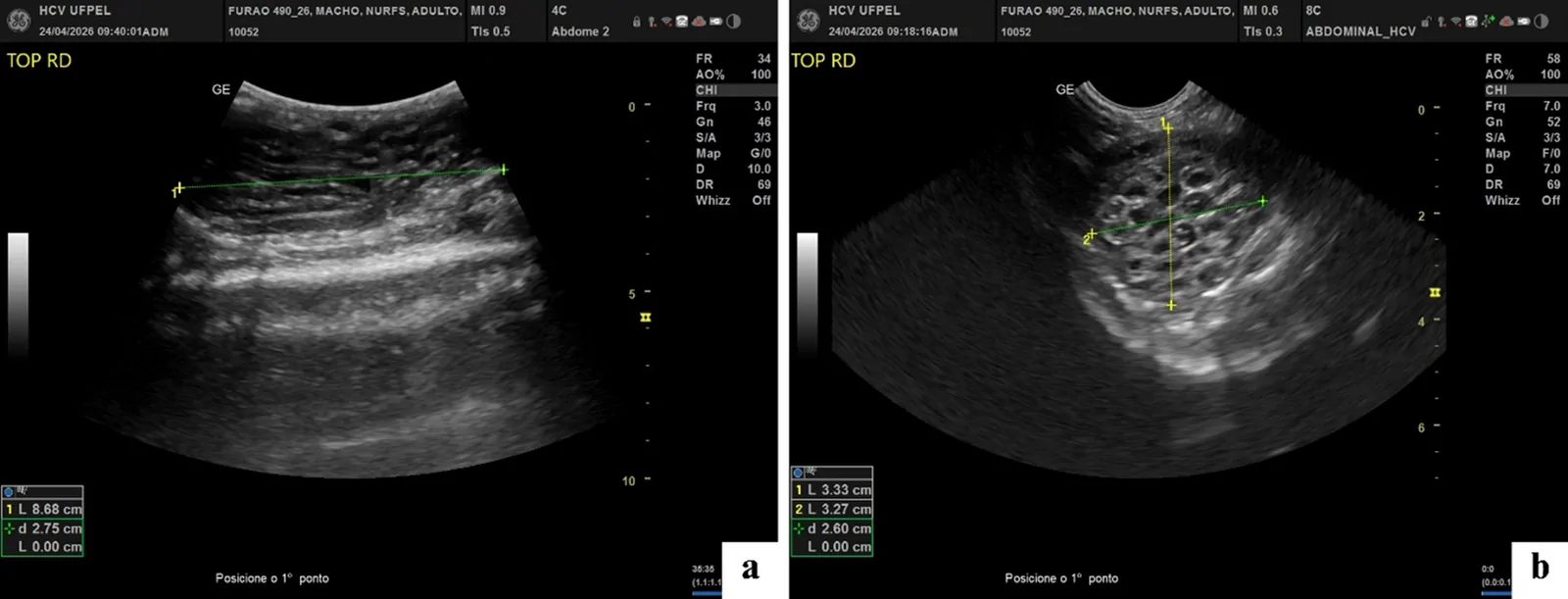
B-mode ultrasonographic images showing (**a**) multiple tubular structures (longitudinal section) (**b**) circular structures (transverse section) with hyperechoic margins and an anechoic center, with no visible renal parenchyma

Abdominal ultrasonography revealed multiple tubular structures in longitudinal section and circular structures in transverse section in the right kidney region. These structures had hyperechoic margins and an anechoic center, with an overall dimensions of 8.68 × 3.27 × 3.33 cm (length × height × width), and were consistent with *D. renale* specimens (Fig. 2). No residual renal parenchyma was identified.

**Fig. 3:**
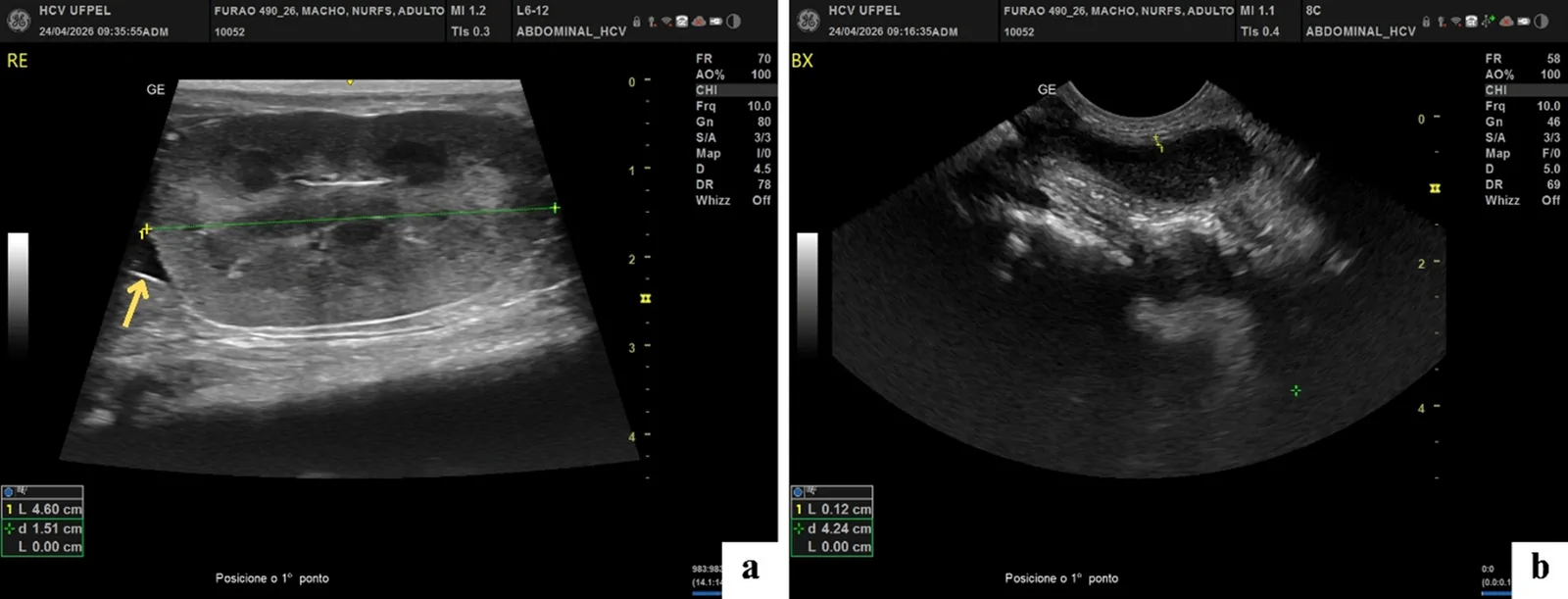
B-mode ultrasonographic images showing (**a**) an enlarged left kidney (4.60 cm), increased cortical echogenicity, and a small anechoic focus consistent with free fluid in the cranial region (arrow) (**b**) the urinary bladder is minimally distended, with a wall of normal thickness and multiple floating echogenic foci consistent with sediment within its lumen

The left kidney measured approximately 4.60 cm, consistent with renal enlargement compared with the measurements reported by Augusti et al. (2019) for *G. cuja* (2.4–3.5 cm). The kidney had regular contours, mildly reduced corticomedullary distinction, and increased cortical echogenicity. The urinary bladder was minimally distended, with a wall of normal thickness (0.12 cm), anechoic contents, and floating echogenic foci consistent with sediment. A small amount of anechoic free abdominal fluid, too small to allow ultrasound-guided sampling, was observed cranial to the left kidney (Fig. 3). No free parasites were identified within the abdominal cavity.

During hospitalization, complete blood counts and serum biochemical analyses were performed at three time points: the first laboratory evaluation on day 14 after admission, the postoperative evaluation on day 40, and the pre-release evaluation on day 45. The results are shown in Table 1.

**Table 1:** Serial hematologic and serum biochemical parameters of an adult male lesser grison (*Galictis cuja*) with renal dioctophymosis at three time points

Parameter	Unit	Day 14	Day 40	Day 45
Complete Blood Count				
Red Blood Cells	×10⁶/µL	4.44	5.63	6.12
Hemoglobin	g/dL	9.5	12.3	12.8
Hematocrit	%	26.5	34.1	38.7
MCV	fL	59.7	60.5	63.2
MCHC	%	36	36	33.1
Platelets	×10³/µL	601	620	457
White Blood Cells	/µL	11,500	25,900	13,400
Neutrophils	/µL	9,660	16,575	8,174
Band neutrophils	/µL	0	0	0
Lymphocytes	/µL	1,380	2,295	2,680
Monocytes	/µL	230	2,040	0
Eosinophils	/µL	230	4,590	2,546
Basophils	/µL	0	0	0
Total protein	g/dL	7.4	8	8.2
Serum biochemistry				
Albumin	g/dL	3	2.7	3.2
Alkaline phosphatase	U/L	32	40	28
Alanine aminotransferase	U/L	195.5	95.6	106.7
Aspartate aminotransferase	U/L	139	86	95
Creatinine	mg/dL	0.21	0.44	0.24
Gamma-glutamyl transferase	U/L	18	19	-
Urea	mg/dL	65	102	71

*MCV*, mean corpuscular volume; *MCHC*, mean corpuscular hemoglobin concentration

**Fig. 4:**
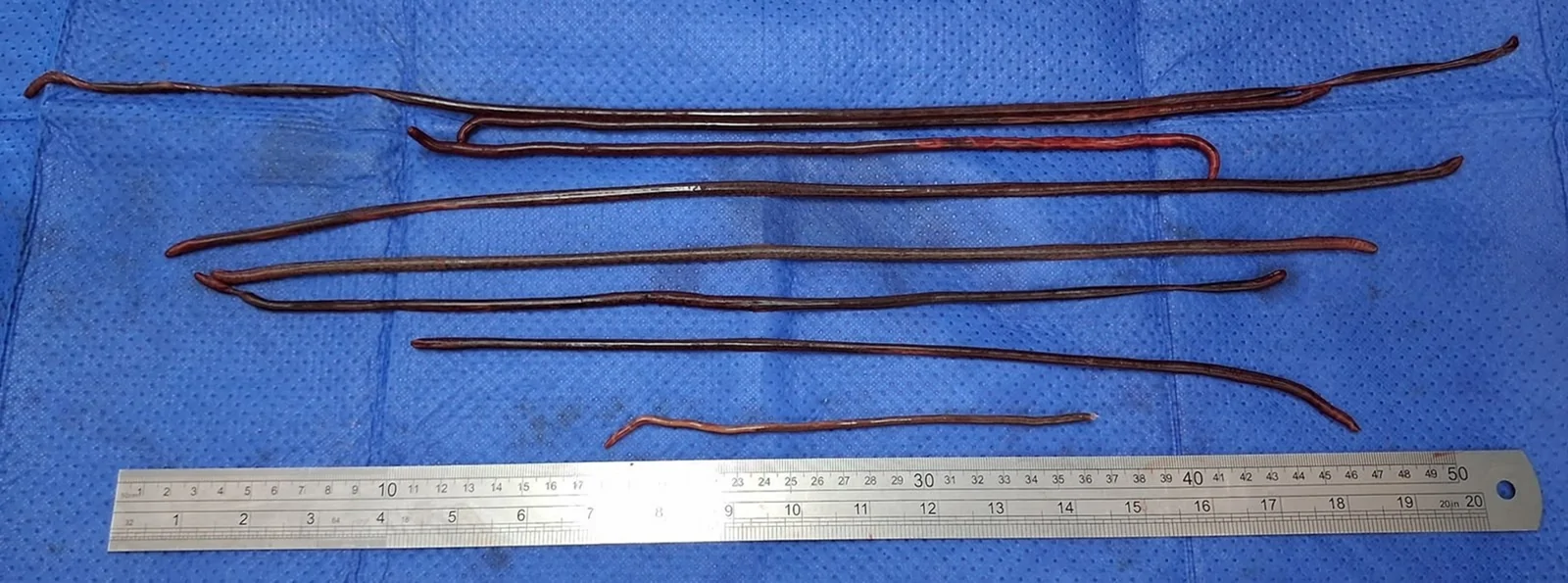
Eight adult *Dioctophyme renale* specimens, comprising seven females (larger) and one male (smaller; the arrow indicates the posterior end of the male, characterized by the bell-shaped copulatory bursa), removed from the right kidney of *Galictis cuja* following nephrectomy

Based on these findings, a total right nephrectomy was performed. Eight adult *D. renale* specimens were identified in the kidney, comprising seven females and one male (Fig. 4). The excised kidney was submitted for pathological examination. Grossly, the right kidney had an irregular capsule, no identifiable renal parenchyma, a brownish color, and a firm cut surface. Microscopic examination revealed osseous and squamous epithelial metaplasia of the renal capsule, with no viable glomeruli or renal tubules present.

During the postoperative period, analgesia consisted of meloxicam (0.1 mg/kg, subcutaneously, every 24 h), dipyrone (25 mg/kg, subcutaneously, every 12 h), and tramadol (5 mg/kg, intramuscularly, every 12 h). Antimicrobial therapy consisted of amoxicillin trihydrate (20 mg/kg, subcutaneously, every 12 h). Recovery was uneventful. The skin sutures were removed 10 days after surgery, allowing the animal to recover and return to the wild.

## Discussion and conclusions

*Galictis cuja* has been described as a definitive host of *D. renale*, with adult parasites reported in the kidneys of free-ranging individuals (Barros et al. 1990; Pesenti et al. 2012; Zabott et al. 2012; Pedrassani et al. 2017). In these hosts, adult parasites reproduce within the kidneys and shed eggs in the urine (Kommers et al. 1999; Pedrassani and Nascimento 2015). Infection requires an annelid as an intermediate host, while fish, amphibians, and reptiles have been described as paratenic hosts (Pedrassani et al. 2009; Mascarenhas and Müller 2015; Mascarenhas et al. 2018, 2019). *Galictis cuja* is a predominantly carnivorous and opportunistic species whose diet includes mammals, birds, reptiles, amphibians, fish, invertebrates, and occasionally plant material (Yensen and Tarifa 2003; Cheida et al. 2011; Queiros et al. 2024; Rodrigues et al. 2025). Therefore, the ingestion of paratenic hosts may represent an important route of exposure of this species to infective *D. renale* larvae.

Fecal parasitological examination revealed capillariid-type eggs, *D. renale* eggs, Strongylida-type eggs, and unsporulated coccidian oocysts morphologically compatible with *Cystoisospora* spp. Identification of the coccidian oocysts was based on their morphological characteristics and dimensions. However, the observed oocysts were not fully sporulated; therefore, the morphological features required for definitive genus-level identification were not fully developed. Although *Cystoisospora* spp. was considered the most likely identification based on the observed characteristics, differentiation from other morphologically similar coccidia, including *Isospora* spp., cannot be considered definitive based solely on these findings. Given that the sample was collected from the enclosure and the examination was neither repeated nor supplemented by other techniques, these results were interpreted as a screening; it was not possible to confirm patent infections or establish their clinical relevance. Furthermore, considering that *G. cuja* is a free-ranging carnivore, the possibility of pseudoparasitism cannot be entirely ruled out, and the detection of parasitic elements in the feces should be interpreted with caution, as it does not constitute definitive evidence of an established infection in the lesser grison (*G. cuja*). Additionally, although *G. cuja* may harbor a diverse helminth fauna, knowledge regarding its parasites remains limited (Corrêa et al. 2016). The detection of *D. renale* eggs in the fecal sample was interpreted as probable urine contamination.

On urinalysis, mild proteinuria and sediment containing erythrocytes, leukocytes, and renal epithelial cells may have been consistent with urinary tract injury and inflammation associated with the action of the parasite, although these findings are nonspecific (Conte et al. 2021). Bacteriuria, in particular, was not interpreted as evidence of urinary tract infection because the urine was obtained by spontaneous urination. In contrast, the high concentration of yellowish-brown, thick-shelled, rough-surfaced, bipolar-operculated eggs in the urine sediment, together with the ultrasonographic and surgical identification of the parasites, confirmed patent *D. renale* infection. The detection of eggs in urine is an accessible diagnostic method; however, its sensitivity is limited, and false-negative results may occur in infections involving only males, immature females, or parasites located at ectopic sites (Capella et al. 2024).

Abdominal ultrasonography is an important tool for diagnosing dioctophymosis because it localizes parasites and assesses the extent of renal involvement. Circular structures in transverse section and tubular or band-like structures in longitudinal section, with hyperechoic walls and an anechoic center, are characteristic of *D. renale* within the kidney (Rahal et al. 2014; Zadeh et al. 2025). In dogs, this method has shown high sensitivity, including in cases without urinary egg shedding (Capella et al. 2024). In the present case, the echogenic foci observed within the urinary bladder lumen were consistent with urinary sediment, the composition of which was determined by urinalysis, revealing cellular elements and numerous *D. renale* eggs. These findings confirmed patent infection and the presence of at least one adult female (Valls Sanchez et al. 2019; Moosavian et al. 2024). The small amount of free fluid cranial to the left kidney was considered a nonspecific finding, possibly related to trauma, but could not be characterized because of its low volume (Boysen et al. 2004). The absence of free parasites should be interpreted with caution because ultrasonography is less effective at detecting ectopic specimens (Rahal et al. 2014). Enlargement of the left kidney was suggestive of compensatory hypertrophy and should be interpreted together with the clinical course and serial biochemical findings (Churchill et al. 1999; Mesquita et al. 2014).

No pharmacological antiparasitic treatment has proven efficacy against dioctophymosis; therefore, surgical removal of the parasites remains the primary therapeutic approach (Santos et al. 2022). The choice between nephrotomy and nephrectomy depends mainly on the degree of preservation of the renal parenchyma (Freitas et al. 2018; Pizzinatto et al. 2019). In the present case, the absence of identifiable renal parenchyma in the right kidney and the advanced structural damage justified nephrectomy. Hypertrophy of the left kidney and the absence of progressive increases in urea and creatinine concentrations during serial evaluations were consistent with compensation by the contralateral kidney (Mesquita et al. 2014).

Pathological examination confirmed advanced destruction of the parasitized kidney, characterized by the absence of viable glomeruli and tubules and by osseous and squamous metaplasia of the renal capsule. Dioctophymosis may cause progressive loss of renal parenchyma through the mechanical action of the parasites, combined with a chronic inflammatory response and replacement of renal tissue by fibrous or metaplastic structures (Nakagawa et al. 2007; Russo et al. 2022). The adult worm also exhibits hematophagous and histophagous behavior, feeding on blood and renal parenchyma and generally causing extensive destruction of the affected kidney (Zadeh et al. 2025). In the present case, these findings indicated irreversible renal damage and supported the decision to perform complete surgical removal of the affected organ.

The absence of species-specific hematological and biochemical reference intervals for *G. cuja* limits the classification of the results as increased or decreased. Therefore, an intraindividual longitudinal assessment was adopted, with descriptive comparison of the three serial measurements obtained from the same animal, an approach that is useful when population-based reference intervals are unavailable (Walton 2012). The erythrogram showed progressive increases in erythrocyte count, hemoglobin concentration, and hematocrit throughout rehabilitation. The total leukocyte count showed a transient increase during the second evaluation, performed nine days after nephrectomy, followed by a decrease at the third sampling, mainly reflecting changes in neutrophil, monocyte, and eosinophil counts. In this case, the hematological variations may have been jointly influenced by trauma resulting from the vehicle collision, the inflammatory process associated with parasitism, the surgical procedure, and clinical recovery; therefore, they could not be attributed exclusively to *D. renale* infection.

On serial biochemical evaluation, alanine aminotransferase (ALT) and aspartate aminotransferase (AST) activities were higher at the first sampling and decreased in subsequent evaluations. Urea and creatinine concentrations showed transient increases during the second evaluation, followed by decreases during the third, with no progressive, concurrent increase in these markers. This pattern, together with the compensatory hypertrophy observed in the left kidney, was consistent with functional compensation by the contralateral kidney. In dogs with unilateral dioctophymosis, changes in serum urea and creatinine concentrations may be mild or absent because of compensation by the nonparasitized kidney, both before and after nephrectomy (Mesquita et al. 2014; Valle et al. 2022).

Considering the feeding habits of *G. cuja* and the occurrence of adult specimens within the kidney, this species may contribute to the maintenance and dispersal of *D. renale* in wild environments. However, the domestic and sylvatic cycles should not be regarded as entirely independent. Genetic studies have demonstrated shared haplotypes between domestic and wild carnivores and found no parasite population structuring according to host species, indicating that *D. renale* circulates among different host species (Arce et al. 2025). Increased contact between domestic animals and wildlife, driven by habitat fragmentation and urbanization, may create further opportunities for transmission of the parasite (Assis-Silva et al. 2025).

The origin of the animal is also epidemiologically relevant because it was found in an urban area of Capão do Leão, a municipality where *D. renale* has previously been identified in a wild carnivore, *Leopardus geoffroyi* (Trindade et al. 2018), and third-stage larvae have been reported in a snake, *Philodryas patagoniensis*, which may act as a paratenic host (Mascarenhas et al. 2018). In the neighboring municipality of Pelotas, infection has been documented in domestic dogs and cats (Rappeti et al. 2017), free-roaming dogs (Brunner et al. 2022), and through the identification of parasite eggs in animal urine and urban soil (Perera et al. 2017). Taken together, these findings demonstrate the circulation of different developmental stages of *D. renale* among domestic and wild hosts and in the environment, supporting the local maintenance of the parasite’s life cycle in the region.

A limitation of this report is that species identification of the parasite was based on morphological and clinical findings, including the characteristic morphology of eggs detected in urine, ultrasonographic findings, and gross examination of the adult nematodes recovered during nephrectomy, without molecular confirmation. Although these findings strongly supported the diagnosis of *D. renale* infection, molecular characterization would have strengthened species-level identification and could have provided additional information on genetic variation. Future studies should consider PCR and sequencing of adult worms or egg-derived DNA when suitable material is available.

In conclusion, *D. renale* infection should be considered a differential diagnosis in wild mustelids originating from endemic regions, even in the absence of laboratory abnormalities suggestive of renal impairment. The combination of urinalysis, abdominal ultrasonography, and clinical assessment was essential for diagnosis and therapeutic planning. Nephrectomy was effective given the complete destruction of the parasitized kidney, allowing the animal to recover and return to the wild.

## Data Availability

No datasets were generated or analysed during the current study.
